# Oxytocin effects on amygdala reactivity to angry faces in males and females with antisocial personality disorder

**DOI:** 10.1038/s41386-023-01549-9

**Published:** 2023-03-20

**Authors:** Haang Jeung-Maarse, Mike M. Schmitgen, Ruth Schmitt, Katja Bertsch, Sabine C. Herpertz

**Affiliations:** 1 Department of Psychiatry and Psychotherapy, Evangelisches Klinikum Bethel (EvKB), Bielefeld University, Bielefeld, Germany; 2 Department of General Psychiatry, Heidelberg University Hospital, Heidelberg, Germany; 3 Department of Psychology, Ludwig Maximilian University of Munich, Munich, Germany

**Keywords:** Psychiatric disorders, Human behaviour, Amygdala

## Abstract

The amygdala is a key region in current neurocircuitry models of reactive aggression as it is crucially involved in detecting social threat and provocation. An increased amygdala reactivity to angry faces has been reported in aggression-prone individuals and the neuropeptide oxytocin (OT) could dampen anger-related amygdala reactivity in a number of mental disorders. One example is the antisocial personality disorder (ASPD) which has so far only been studied in limited numbers. To address the question whether OT can normalize amygdala hyperreactivity to emotional faces, we conducted a functional magnetic resonance imaging experiment with 20 men and 18 women with ASPD and 20 male and 20 female healthy control (HC) participants in a double-blind, randomized, placebo (PLC)-controlled within-subject design. Participants were exposed to an emotion classification task (fearful, angry, and happy faces) after receiving an intranasal dose (24 IU) of synthetic OT or PLC. We found OT to attenuate right amygdala hyperactivity to angry faces in participants with ASPD to such an extent that the intensity of amygdala activity in the ASPD group in the OT condition decreased to the level of amygdala activity in the PLC condition in the HC group. There was also a trend that OT effects were generally larger in women than in men. These findings suggest that OT differentially modulates the amygdala following social threatening or provoking cues in dependence of psychopathology (ASPD vs. HC) and sex (male vs. female). Particularly female ASPD patients could benefit from OT in the treatment of reactive aggression.

## Introduction

Reactive aggression is often defined as response to perceived social threat, provocation, frustration, or rejection [1]. Unlike instrumental aggression, reactive aggression is usually associated with anger [2]. The amygdala has been identified as a key region in the brain’s threat circuitry [3] in both rodent [4] and human lesion studies [5]. Correspondingly, amygdala activity correlated positively with aggressive responding to provocation in healthy individuals [6–8]. However, findings regarding amygdala responses to anger- and aggression-eliciting stimuli in aggression-prone individuals compared to healthy individuals are inconsistent [9, 10]. For instance, violent offenders showed increased amygdala activity in response to angry faces [11–13], anger-inducing videos [14], and provocation followed by more aggressive monetary-reinforced responding [15]. By contrast, threat and anger provocation did not relate to amygdala hyperresponsivity in other studies with violent offenders [8, 16, 17]. There was even a reduced amygdala activity when adolescents with conduct disorder (CD) processed angry faces [18].

One neuromodulator of the amygdala is oxytocin (OT) [19] which regulates prosocial but also antisocial behaviors [20]. Hence, the social salience hypothesis of OT [20] describes the overarching role of OT in regulating the salience of social cues, especially signaling threat and thus promoting active defensive behaviors [21]. For instance, OT elevated monetary-reinforced aggression in healthy men [22]. At the same time, OT attenuated amygdala reactivity to angry faces [23–25], decreased amygdala activation during threat approach [26] and facilitated threat-specific amygdala desensitization [27]. These findings are subsumed under the social anxiolytic effects of OT and might have clinical implications for aggression-prone individuals. Yet, it is unclear whether these findings in predominantly male, healthy, non-aggressive individuals can be transferred to aggression-prone individuals [28]. There is only one neuroimaging study which tested OT’s effect on threat-specific amygdala reactivity in a clinical sample: In women with borderline personality disorder (BPD), OT normalized increased amygdala activation in response to angry faces [29].

While reactive aggression can be present in a number of mental disorders, it is central to the antisocial personality disorder (ASPD) [30]. Currently, neither psychotherapy nor pharmacotherapy effectively address reactive aggression in ASPD [31]. While OT might be a promising agent in ASPD [28], there are only two studies that examined OT effects on reactive aggression in ASPD [32, 33]. In a preliminary study with six individuals with ASPD, intranasal OT did not influence monetary-reinforced aggressive responding [32]. In contrast, we could show that OT normalized shorter response latencies in the classification of angry faces in ASPD in comparison to healthy controls [33]. However, our prior study measured only behavioral outcomes and the small sample size of the mixed-sex group did not allow to study sex-specific differences in the OT effects. Meanwhile, sex-specific effects of OT on neural correlates have been reported in healthy individuals [34–36].

The present study examined the effect of a single dose of intranasal OT on amygdala reactivity to emotional facial expressions in men and women with ASPD in comparison to healthy men and women in a double-blind, randomized, placebo-controlled within-subject design. We used blood oxygenation level-dependent (BOLD) fMRI coupled with an emotional face classification task which has been established in the investigation of amygdala activation [37] and its oxytocinergic modulation in response to early phase recognition of fearful, angry, and happy faces while controlling for the initial fixation on eyes or mouth in healthy [38] and clinical [29] subjects. Behavioral outcomes of this task are proportion of correct responses and response latencies as the well-established combination of these two measurements gives particular insight in the cognitive ability to correctly classify facial emotions together with the partly unconscious processing times of facial emotions [39]. Based on predictions about oxytocinergic modulation of the amygdala during facial emotion classification, we employed an a-priori region of interest (ROI) approach. We investigated whether (1) individuals with ASPD differed from healthy controls in behavioral and amygdala responses to fearful, angry, and happy faces, (2) whether these differences were normalized by OT, and (3) whether there were sex-specific differences in OT-modulated neural effects.

## Materials and Methods

### Participants

Participants were 20 men and 18 women with ASPD and 20 male and 20 female healthy control (HC) participants aged 18–30 years (for details, see Supplementary Materials and Methods). ASPD participants had to fulfil the DSM-IV, respectively DSM-5, criteria for ASPD (according to the International Personality Disorder Examination (IPDE)) and were recruited at the Department of General Psychiatry at the University Hospital of Heidelberg, through local probation services, and through institutions offering anti-aggression trainings. HC participants were age- and IQ-matched and recruited through advertisement (black boards, online platforms, website of University Hospital of Heidelberg).

Exclusion criteria for all participants were an intelligence quotient (IQ) lower than 80, insufficient German language skills, pregnancy, breast feeding, claustrophobia, any current medication (except oral contraceptives and levothyroxine), past cranio-cerebral injuries, and somatic, endocrine, or neurological diseases. Additional exclusion criteria for ASPD participants were autism spectrum disorder, lifetime diagnoses of schizophrenia or bipolar disorder, and current alcohol or drug dependence. Additional exclusion criteria for HC participants were any lifetime or current mental disorder or personality disorder and any psychological/psychiatric treatment assessed by the Structured Clinical Interview for DSM-IV (SCID-I) and antisocial behavior assessed by the International Personality Disorder Examination (IPDE). Four subtests of the Wechsler Adult Intelligence Scale were used to estimate intelligence [40]. Other clinical screening measures involved the Self-Report Psychopathy Scale [41] (SRP) and the State-Trait Anxiety Inventory [42] (STAXI; for details, see Supplementary Materials and Methods).

The study was approved by the local ethics committee of the Faculty of Medicine, Heidelberg University, and conducted in accordance with the Declaration of Helsinki. All participants gave written informed consent and were financially remunerated for their participation.

### Study design

The study was conducted with a randomized, double-blind, placebo-controlled within-subject design. After an extensive telephone screening, participants took part at an initial diagnostic assessment (including psychiatric evaluation and symptom/personality questionnaires). They were then invited to two experimental assessments, scheduled four weeks apart, at which they underwent functional magnetic resonance imaging (fMRI). The order of the drug administration was randomized by an independent pharmacist providing a computerized simple randomization list. Participants were instructed to abstain from nicotine, food, and drinks except water for 2 h before the experiment. Upon arrival, urine drug tests had to be negative except from tetrahydrocannabinol (THC) due to its dissemination in the general population and particularly in participants with ASPD [43] (drug tests, see Supplementary Material and Methods). In female participants, a urine pregnancy test was conducted and blood was drawn for progesterone assays (progesterone levels, see Supplementary Materials and Methods). Saliva samples were collected pre-administration, and salivary OT was quantified by radioimmunoassay performed by an external lab (OT levels, see Supplementary Materials and Methods). Before each scan, participants were given practice trials outside the scanner. Under the supervision of an experimenter, participants self-administered either OT (24 IU) or PLC 45 min before the fMRI scan (for details, see Supplementary Materials and Methods).

### fMRI task

We used the same emotion classification task as in prior studies [29, 33, 44, 45]. As previously described, the emotion classification task [37] followed a 3 × 2 design (facial emotions at full intensity: fearful, angry, happy; regions for initial eye gaze fixation: eyes, mouth; for details, see [33]). Briefly, participants were instructed to classify the facial emotion of each face as accurately and quickly as possible by pressing a corresponding button. We presented the faces with either the eyes or mouth at the location of a former fixation cross.

### fMRI data acquisition and Region-of-interest (ROI) analysis

We used a 3 T Siemens Trio scanner to record images and applied a T2* weighted reverse echo planar imaging (EPI) sequence for blood-oxygen-level-dependent (BOLD) functional images using an amygdala-sensitive sequence (for details, see Supplementary Materials and Methods). Images were preprocessed and analyzed using SPM8 (for details, see Supplementary Materials and Methods). Final analysis used a region-of-interest (ROI) approach. Anatomically-defined masks for the amygdala were created using the Talaraich atlas in the WFU PickAtlas tool [46]. Parameter estimates were extracted using the MARSBAR toolbox for SPM [47].

### Statistical analysis

Statistical analysis was performed using SPSS 25.0. The effects of OT on task performance and amygdala activation were assessed using repeated measured analyses of covariance (ANCOVAs) with the between-subject factors group (ASPD, HC) and sex (female, male), and within-subject factors condition (placebo, oxytocin), emotion (fearful, angry, happy), initial fixation (eyes, mouth), and hemisphere (left, right). We used Huynh-Feldt correction in case of violations of the assumption of sphericity. For significant results, post-hoc tests were performed by Dunn’s multiple comparison procedure including Bonferroni correction for multiple testing. All statistical analyses were performed two-tailed with a level of significance of *p* < 0.05. Relationships between amygdala activation and behavioural/psychological/hormonal variables were then assessed using correlation analyses.

## Results

### Do individuals with ASPD differ from healthy controls in behavioral and amygdala responses to facial emotional expressions?

To examine whether individuals with ASPD and healthy controls differ in proportion of correct responses, response latencies, and amygdala activation in response to facial emotion expressions, we run group by emotion by initial fixation ANOVAs for the placebo (PLC) condition (for amygdala responses, this analysis also included the repeated-measures factor hemisphere). To answer our first research question, we will report effects including the factor group. To keep the results clear, we do not report non-significant effects of the other factors.

### Proportion of correct responses

Individuals with APSD tended to make more errors than healthy controls in the emotion classification task (*F*(1, 70) = 3.75, *p* = 0.057, *η²* = 0.051). Descriptively, individuals with ASPD were worse than healthy controls at classifying happy and fearful faces, while performances between groups did not differ for angry faces (group by emotion interaction: *F*(2, 140) = 2.36, *p* = 0.098, *η²* = 0.033). Furthermore, there was a significant group by fixation interaction (*F*(1, 73) = 4.03, *p* = 0.049, *η²* = 0.028). Post-hoc tests showed that the ASPD group was better at classifying emotions when the eye region was initially fixated (*p* < 0.050). In comparison, there was no difference in the proportion of correct responses in dependence of the initial fixation of facial region in the HC group (*p* > 0.050). HC were more precise than individuals with ASPD when the initial fixation was on the mouth (*p* < 0.010) and the eye region (*p* < 0.050). The group by emotion by fixation interaction was not significant (*F*(1.96, 138.03) = 1.09, *p* = 0.339, *η²* = 0.015).

### Response latencies

Overall, there was no significant group effect on response latencies (*F*(1, 70) = 0.23, *p* = 0.631, *η²* = 0.003). There was a significant group by emotion interaction (*F*(2, 140) = 5.58, *p* = 0.005, *η²* = 0.005) (Fig. 1.1). Post-hoc tests revealed that, in each group, participants were faster in classifying happy than fearful and angry faces (all *p*’s < 0.010). More interestingly, individuals with ASPD responded faster to angry faces than HC (*p* < 0.010). Neither the effect of group by fixation (*F*(1, 73) = 1.69, *p* = 0.198, *η²* = 0.024) nor the group by emotion by fixation interactions (*F*(1.89, 132.22) = 1.99, *p* = 0.144, *η²* = 0.028) were statistically significant.

**Fig. 1:**
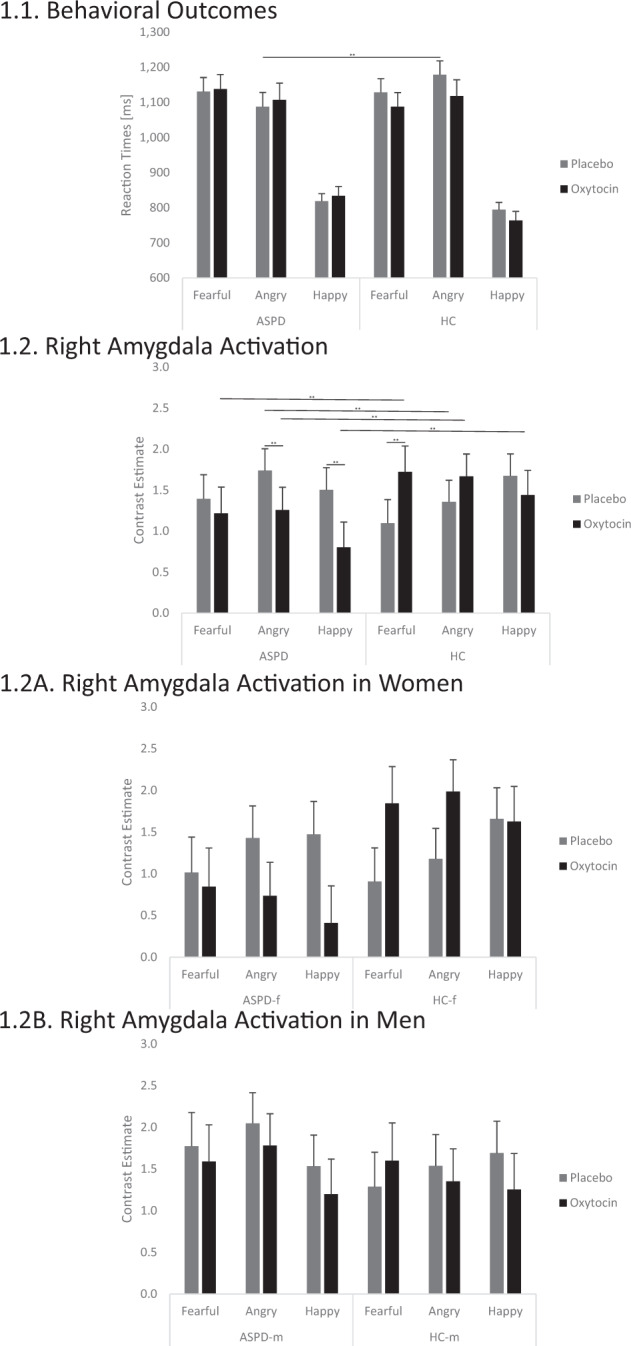
Comparison of reaction times and right amygdala activation after placebo (PLC) and oxytocin (OT) administration in individuals with antisocial personality disorder (ASPD, ***N*** = 38)) compared to healthy control (HC, ***N*** = 40) participants. 1.1. Comparison of reaction times between individuals with ASPD and HC participants as a function of sex (female, male), facial expression (fearful, angry, happy) and initial presentation of facial region (eyes, mouth) in the PLC) and OT condition. Under PLC, there was a significant group by emotion interaction (*F*(2, 140) = 5.58, *p* = 0.005, *η²* = 0.005). Post-hoc tests revealed that, in each group, participants were faster in classifying happy than fearful and angry faces (all *p*’s < 0.010). More interestingly, individuals with ASPD responded faster to angry faces than HC (*p* < 0.010). Error bars indicate standard error of the mean. Abbreviations: ms, milliseconds. **p < 0.010. 2 Comparison of right amygdala activation between individuals with ASPD and HC participants as a function of sex (female, male), facial expression (fearful, angry, happy) and initial presentation of facial region (eyes, mouth) in the PLC and OT condition. There was a significant threefold interaction effect of group by condition by emotion by hemisphere (*F*(2, 146) = 4.27, *p* = 0.016, *η²* = 0.055). In post-hoc tests for the right amygdala, the ASPD group showed a higher activation than the HC group when angry faces were shown under PLC (*p* < 0.010). Under OT, the ASPD group had a lower activation than the HC group at presentation of fearful, angry, and happy faces (all *p*’s < 0.010). This group differences could be accounted by a lower right amygdala activation for angry and happy faces in the ASPD group under OT compared to PLC and a higher right amygdala activation for fearful faces in the HC group under OT compared to PLC (all *p*’s < 0.010). Essentially, the group difference in right amygdala activation for angry faces could be reversed under OT: The ASPD group under OT showed a similarly low right amygdala activation like the HC group under PLC (*p* > 0.050) and the HC group under OT showed a similarly high right amygdala activation like the ASPD group under PLC (*p* > 0.050). Error bars indicate standard error of the mean. Abbreviations: ** *p* < 0.010. 1.2 **A** Right amygdala activation in women with and without ASPD. 1.2 **B** Right amygdala activation in men with and without ASPD. There was a statistically not significant trend for the interaction effect of group by sex by condition by emotion by hemisphere (*F*(2, 146) = 2.48, *p* = 0.087, *η²* = 0.033). Descriptively, OT affected women of the ASPD and HC groups more than men of the ASPD and HC groups.

### Amygdala activation

We found a significant group by emotion by fixation by hemisphere interaction (*F*(2, 146) = 3.46, *p* = 0.034, *η²* = 0.045). Post-hoc tests revealed significant differences in the right amygdala when the mouth region was initially fixated: Individuals with ASPD showed stronger right amygdala activations for angry vs. fearful faces (*p* < 0.010), but no significant differences between angry and happy or fearful and happy faces (all *p*’s > 0.050). In the HC group, happy faces resulted in stronger right amygdala activation than fearful and angry faces (all *p*’s < 0.010), while there was no difference between fearful and angry faces (*p* > 0.050). When the mouth region was initially fixated, group comparisons showed stronger right amygdala activation for individuals with ASPD vs. HC for angry faces (*p* < 0.010), but no significant differences for fearful or happy faces (all *p*’s > 0.050). Neither the main effect of group (*F*(1, 73) = 0.21, *p* = 0.648, *η²* = 0.003) nor the interactions of group by emotion (*F*(2, 146) = 0.79, *p* = 0.455, *η²* = 0.011), group by fixation (*F*(1, 73) = 0.73, *p* = 0.787, *η²* = 0.021), and group by emotion by fixation (*F*(2, 146) = 0.44, *p* = 0.644, *η²* = 0.006) were statistically significant. Furthermore, we found a statistically not significant trend for the group by fixation by hemisphere interaction (*F*(1, 73) = 3.61, *p* = 0.062, *η²* = 0.047). Descriptively, the right amygdala activation for emotional faces was higher in the HC group than in the ASPD group when the eyes region was initially fixated.

### Does OT modulate emotion classification and amygdala processing differently in individuals with ASPD and healthy controls?

Next, we were interested in investigating effects of OT on amygdala and behavioral responses in individuals with ASPD as compared to healthy volunteers. Therefore, we conducted group by condition by emotion by fixation ANOVAs (for amygdala responses, the analysis also included the repeated-measures effect of hemisphere). To answer our second research question, we will report whether the factor condition interacted with the significant and trend-level findings in the placebo (PLC) condition.

### Proportion of correct responses

Neither the group by condition (*F*(1, 70) = 1.13, *p* = 0.292, *η²* = 0.016) nor the group by condition by emotion interaction (*F*(1, 70) = 1.23, *p* = 0.295, *η²* = 0.017) nor the interaction effect of group by condition by fixation (*F*(1, 70) = 0.48, *p* = 0.490, *η²* = 0.007) reached statistical significance. Since the proportion of correct responses was generally high (*M* = 8.8, *SE* = 0.01 in the ASPD group, *M* = 0.91, *SE* = 0.01 in the HC group), we cannot dismiss a ceiling effect.

### Response latencies

The interaction effect of group by condition by emotion was not significant (*F*(1.78, 124.53) = 0.51, *p* = 0.578, *η²* = 0.007) (Fig. 1.1).

### Amygdala activation

The interaction effect of group by condition by hemisphere by emotion by fixation was not significant (*F*(2,146) = 1.69, *p* = 0.189, *η²* = 0.023). However, we found the threefold interaction effect of group by condition by emotion by hemisphere to be significant (*F*(2, 146) = 4.27, *p* = 0.016, *η²* = 0.055) (Fig. 1.2). In post-hoc tests for the right amygdala, the ASPD group showed a higher activation than the HC group when angry faces were shown under PLC (*p* < 0.010). Under OT, the ASPD group had a lower activation than the HC group at presentation of fearful, angry, and happy faces (all *p*’s < 0.010). This group differences could be accounted by a lower right amygdala activation for angry and happy faces in the ASPD group under OT compared to PLC and a higher right amygdala activation for fearful faces in the HC group under OT compared to PLC (all *p*’s < 0.010). Essentially, the group difference in right amygdala activation for angry faces could be reversed under OT: The ASPD group under OT showed a similarly low right amygdala activation like the HC group under PLC (*p* > 0.050) and the HC group under OT showed a similarly high right amygdala activation like the ASPD group under PLC (*p* > 0.050). Further, the post-hoc tests showed no group differences in the left amygdala activation under PLC. Under OT, the ASPD group had a lower left amygdala activation in response to fearful and happy faces than the HC group (all *p*’s < 0.010). This effect was driven by the ASPD group with lower left amygdala activation in response to happy faces under OT compared to PLC (*p* < 0.010) and the HC group with higher left amygdala activation in response to any facial emotion under OT compared to PLC (all *p’s* < 0.010). There were no group differences under OT when angry faces were shown (*p* > 0.050).

### Is there an influence of sex on OT modulation in individuals with ASPD compared to healthy controls?

In order to explore the sex-specific effect of OT on group differences, we calculated the additional sex-, group-, and condition-related interaction effects. We will only report significant and trend-level significant results.

### Behavioral outcomes

We did not find significant or trend-level interaction effects of sex.

### Amygdala activation

We found a statistically not significant trend for the interaction effect of group by sex by condition by emotion by hemisphere (*F*(2, 146) = 2.48, *p* = 0.087, *η²* = 0.033) (Fig. 1.2A and 1.2B). Descriptively, OT affected women of the ASPD and HC groups more than men of the ASPD and HC groups. Particularly in the right amygdala, the activation for angry (*M* = 0.74, *SE* = 0.40 vs. *M* = 1.43, *SE* = 0.39 vs.) and happy (*M* = 0.41, *SE* = 0.45 vs. *M* = 1.47, *SE* = 0.40) faces were lower under OT than under PLC in the female ASPD group and activation for fearful (*M* = 1.84, *SE* = 0.44 vs. *M* = 0.91, *SE* = 0.40) and angry (*M* = 1.99, *SE* = 0.38 vs. *M* = 1.18, *SE* = 0.37) faces were higher under OT than under PLC in the female HC group.

### Correlations Between Amygdala Activation and Behavioral Outcomes Following Placebo and Oxytocin Administration

**Fig. 2:**
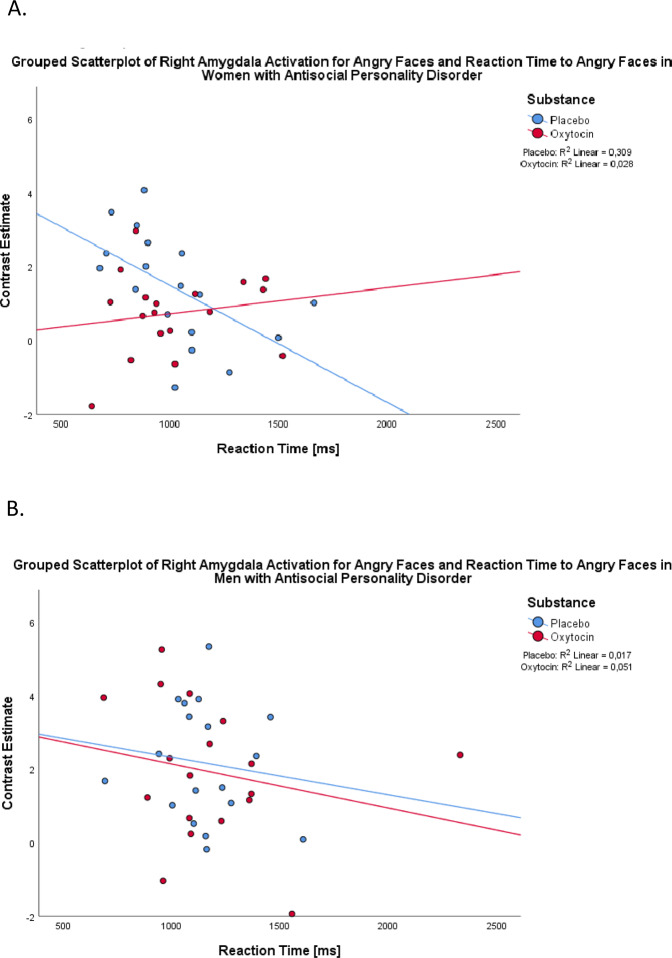
Correlation between right amygdala activation for angry faces and reaction time to angry faces after placebo (PLC) and oxytocin (OT) administration in women and men with antisocial personality disorder (ASPD, ***N*** = 38). **A** The scatterplot depicts the correlation of right amygdala activation for angry faces and reaction time to angry faces in women with ASPD (*N* = 18) under PLC under PLC (*R*^*2*^ = 0.309) and OT (*R*^*2*^ = 0.028). **B** The scatterplot depicts the correlation of right amygdala activation for angry faces and reaction time to angry faces in men with ASPD (*N* = 20) under PLC (*R*^*2*^ = 0.017) and OT (*R*^*2*^ = 0.051).

Faster response latencies to angry faces correlated with increased activation in the left (*r* = −0.506, *p* < 0.050) and right (*r* = −0.556, *p* = 0.050) amygdala activation in women with ASPD in the PLC but not in OT condition (left: *r* = −0.019, *p* > 0.050; right: *r* = −0.034, *p* > 0.050) (for the right amygdala: Fig. 2A). In the other three groups, amygdala activation did not correlate with response latencies to angry faces under neither PLC nor OT (all other correlations |*r* | ≤ 0.400, *p* > 0.050) (as an example, see Fig. 2B for the non-significant correlation between response latencies and right amygdala activation to angry faces in men with ASPD in the PLC vs. OT condition).

## Discussion

Oxytocin (OT) has been proposed to be beneficial to several psychiatric disorders characterized by social dysfunction by modulating the amygdala during facial emotion recognition [48]. As we had previously found OT to normalize facial emotion classification in ASPD [33], we examined whether OT would modulate amygdala activity in ASPD which would provide a neural mechanism to facilitate emotion processing. We found individuals with ASPD to show right amygdala *hyperactivit*y specifically to *angry* faces, relative to healthy control participants. OT attenuated the higher amygdala activity in ASPD participants. Roughly, the intensity of amygdala activity in ASPD in the OT condition decreased to the level of amygdala activity in the PLC condition in healthy controls.

Consistent with our earlier findings obtained with an overlapping sample [33], we observed ASPD participants to show significantly faster responses to angry faces than HC participants under PLC. Normally, the intuitive reaction to angry faces is avoidance [49]. In individuals with an increased experience of anger (trait anger), however, the sight of angry faces was found to lead to approach and attack behavior, as they experience this as provocative [50]. Therefore, aggression might be related to an increased sensitivity to angry facial expressions [51]. In line with this assumption, offenders with ASPD showed a stronger attention bias for violence-related words compared to controls [52]. As before [33], we did not find decreased response latencies at the cost of accuracy in the classification of angry faces in ASPD. However, we found a trend for reduced accuracy in the classification of fearful and happy faces in individuals with ASPD compared to HC participants under PLC. While numerous studies in the youth have reported deficits in emotion recognition in antisocial populations [53], a few studies in adults have not found any deficiency [54] or, respectively, only deficits for facial expressions low in intensity [55, 56]. Since we only employed emotional expressions at full intensities, we cannot dismiss a ceiling effect for the behavioral outcomes. This negative finding is similar to other findings using the same paradigm in healthy [37, 38] and clinical [29] subjects. In addition, while proportion of correct responses may give particular insight in the cognitive ability to correctively classify facial emotions, response latencies may reveal partly unconscious processing times of facial emotions which we examined in particular with this study as we aimed for reflexive processing of facial emotion expressions controlling for the initial fixation on eyes or mouth (see Supplementary Discussion).

Under PLC, we found a stronger right amygdala activation in ASPD vs. HC participants for angry faces when the mouth region was initially presented. This is in line with the executive emotion model, in which increased amygdala responsiveness to threat cues (e.g., angry faces) might lead to reactive aggression [57]. Here, the amygdala is conceptualized as “threat detector” in the salience network (SN) [58], which mediates the detection and integration of behaviorally relevant stimuli [59]. An increased amygdala responsiveness to social threat has been found in female patients with borderline personality disorder (BPD) [29], a condition that is related and often comorbid with ASPD [60]. Also individuals with intermittent explosive disorder, a mental health condition characterized by frequent anger outbursts and aggression, showed an increased amygdala responsiveness to angry faces than healthy controls [12].

After OT administration, we found a decreased right amygdala activity for angry and happy faces in the ASPD group and an increased right amygdala activity for fearful faces in the HC group. Most interesting was the effect of OT on amygdala activation to angry faces: The activation pattern of ASPD and HC were opposite in the OT condition compared to the PLC condition. This group difference in OT effect is in line with the social salience hypothesis [20] which states that the effects of OT are dependent on contextual aspects (such as competitive versus cooperative environment) and baseline individual differences such as sex, personality traits, and degree of psychopathology. OT has been found to reduce amygdala activation to negative emotional stimuli in healthy males [24, 26, 38] and particularly in those participants with enhanced reactivity to social threat such as patients with affective or anxiety-related disorders [29, 61–63]. By contrast, OT has been reported to increase amygdala activation in participants with lower amygdala activation to emotional faces such as patients with autistic spectrum disorder [64, 65]. Whereas the findings of OT effects on anger-related amygdala activation is consistent to the literature, we report that increased amygdala activation under OT in response to fearful stimuli in healthy controls is surprising and in contrast with previous studies in healthy men [24, 66]. Descriptively, this OT effect was driven by healthy women in our study sample. This, in turn, fits to the results that OT increases the salience of social signals by strengthening the sensitivity for fearful faces in the amygdala in women, while OT reduces fear-related amygdala responses in men [36].

There is evidence of hormonal cycle-related OT effects on emotion recognition accuracy in healthy young females [67]. Further, the use of hormonal contraception has been previously found to modulate OT effects [68]. When hormonal levels of estradiol and progesterone were correlated with several neuropsychological parameters in healthy women, there was a significant negative correlation between progesterone level and emotion recognition performance indicating a special influence of progesterone [67]. Our female ASPD and HC groups did not differ in plasma progesterone levels and those taking contraceptives were not excluded from participation in our study (see Supplementary Materials and Methods, Table 2, Results, and Discussion). There is, however, also recent evidence for interactions between OT and estradiol: High endogenous OT and estradiol levels correlated negatively with anxiety levels in highly socially anxious women compared to lowly socially anxious women [69] and high exogenous OT or estradiol but not the combination of the two hormones diminished recognition memory and hippocampal responses in healthy women [70]. In summary, these findings suggest that interactions between OT and sexual hormones are complex as they are dependent on individual OT and sex hormone levels that act partly synergistic and partly antagonistic, brain regions, and individual (psychopathological) characteristics in women.

Although ASPD is prevalent in males and females, most studies have exclusively focused on males [71]. Little is known about phenotypic manifestations and its neurobiological correlates in females with ASPD. In the general population, there were clear sex differences on manifestations of ASPD [72]. Men with ASPD presented more violent antisocial behaviors and women with ASPD reported more aggressive behaviors and irritability. These sex differences might result from different development trajectories associated with different neurobiological underpinnings [71]. In this context, it is interesting that we found an association between response latencies to angry faces and amygdala activity only in ASPD women but not in ASPD men.

One strength of this study is the largest ASPD sample in the fMRI literature [73]. We included male and female participants which is especially important given that sex differences in brain activity during face processing [74, 75] and in neural effects of OT [76] have been reported. Our sample was also well-characterized in terms of comorbid disorders, self-reported psychopathic traits and aggression, and HC groups were matched in age and IQ. We employed a within-subject design with a well-established fMRI task without learning effects [29] which has been shown to target amygdala activation and OT effects in healthy and clinical groups.

Even though the sample size is comparably large, it is limited since we explored up to six-way interactions. Still, we found group effects on amygdala activation which speaks for a validity of our results. Furthermore, we did not have a clinical control group. A significant proportion of the ASPD sample met criteria for psychopathy. However, we decided to be cautious with correlation analyses due to the small sample (9 individuals of 20 male ASPD participants, 7 individuals of 18 female ASPD participants) and the fact that the SRP score has not been a target variable. Yet, the differences between ASPD and psychopathy in emotion classification tasks are not well understood, calling for further studies with larger sample sizes. Second, due to lack of compliance of individuals with ASPD, we resigned from controlling the phase of the menstrual cycle though research indicates that amygdala function in females differs between different phases of their menstrual cycle [77]. We managed to arrange the appointments 4 weeks apart, so that the menstrual phase is likely not to have differed between the testing sessions for each individual. Third, we did not use “neutral” faces as control as we wanted to avoid a hostile interpretation bias, which is the tendency to interpret ambiguous stimuli in a hostile manner, in individuals with ASPD [78]. However, the implementation of emotional faces at full intensity may have led to highly accurate emotion classification and potential ceiling effects in both ASPD and HC groups. Finally, we examined the amygdala as a whole, as it was previously described for this fMRI paradigma [37], while one could also examine subnuclei of the amygdala as different models of the aggression circuitry are discussed [79].

In conclusion, data from this study showed that OT has a specific effect on anger-related amygdala activity in ASPD. OT was found to dampen amygdala activity in response to angry faces that are highly salient for individuals with ASPD and high trait anger especially in ASPD women compared to ASPD men. These findings suggest that attenuating the salience of cues perceived as threatening or provoking, is promising in the treatment of reactive aggression in ASPD patients, particularly when they are females.

## Supplementary information

Supplemental Material 1

Supplemental Material 2
