# Supplementary material for: Oxytocin effects on amygdala reactivity to angry faces in males and females with antisocial personality disorder
Source: Neuropsychopharmacology. 2023 Mar 20;48(6):946–53. doi: 10.1038/s41386-023-01549-9 (PMC10156793; doi:10.1038/s41386-023-01549-9)
Supplement: Supplementary file 1 — Supplemental Material 1 [file 41386_2023_1549_MOESM1_ESM.docx]

**Supplemental Material**

This study was part of a larger EU study on effects of OT in adolescents with CD and adults with ASPD. It has been registered in the German Clinical Trials Register (DRKS) with registration number DRKS00030625. The full trial protocol can be accessed at the DRKS.

**Sample size determination**

According to a sample size plan (GPower^1^), medium-sized population effects (Ω^2^=.10) with a test strength of 1-ß>.80 can be found with a sample of N=80 subjects in a repeated measures design^2^. Thus, the sample should consist of 20 men and 20 women with antisocial personality disorder and 20 healthy men and 20 healthy women. Due to the repeated measurement design, a dropout rate of 20% should be expected; there is also an exclusion of 10% due to measurement artifacts. In conclusion, we aim for a total sample size of N=104 subjects: N=26 men and women each with antisocial personality disorder and N=26 male and female control subjects.

**Materials and Methods**

**Participants**

Initially, 102 participants took part in this study of which 6 participants (3 ASPD men, 2 HC men, 1 HC woman) dropped out not completing the second measurement, 17 participants (5 ASPD men, 4 ASPD women, 3 HC men, 5 HC women) had to be excluded from further analyses because of technical artefacts in the scan, and 1 participant (ASPD woman) was excluded due to an acute neurological finding in the scan. In cases the data preprocessing of a measurement showed motion artefacts in the scan, we aimed at repetition of the measurement affected since we did not expect learning effects^3^. In this way, we could replace otherwise non-evaluable measurements of six individuals (3 ASPD men, 2 HC men, 1 HC women). These measurements took place about one year after the original measurements and were all performed under OT (double-blind administration). Please note that the sample is partially overlapping with our prior study sample^4^ where however no fMRI data were reported. Participants were recruited between August 2014 and February 2018.

*Table 1* provides detailed information about participants’ demographic and clinical characteristics. As shown, the groups did not differ significantly in age and IQ. The ASPD groups scored significantly higher on psychopathy and had a higher proportion of borderline personality disorder (BPD), past major depressive episode (MDE), alcohol abuse, cannabis abuse, multiple substance abuse, and post-traumatic stress disorder (PTSD). In post-hoc tests, women with ASPD had higher comorbidities with BPD, past MDE, and PTSD than men with ASPD (all *p*’s < .05). ASPD participants had significantly higher SRP and the STAXI (sub)scores relative to HC participants. Additionally, the following pairwise differences between the male and female ASPD groups were found in post-hoc analyses: Antisocial behavior was more present in men than women with ASPD (8.05, 95%-CI[1.45, 14.65], p = .01). In contrast, trait anger (6.58, 95%-CI[2.66, 10.49]), anger reaction (3.16, 95%-CI[1.04, 5.28]), and anger temper (3.42, 95%-CI[1.22, 5.63]) was higher in ASPD women than in men with ASPD (all *p*’s ≤ .001) and women with ASPD showed less anger control than men with ASPD (-4.13, 95%-CI[-8.03, -0.23], *p* = .033).

*Table 2* presents salivary oxytocin (OT) levels and urinary cannabis positive tests of all participants, and plasma progesterone levels of female participants. Across groups, salivary OT levels were higher in women than in men (*F*(1, 55) = 6.85, *p* = .011, *η²* = .11). The female HC and ASPD groups did not differ significantly in progesterone levels (*F*(1, 28) = 3.41, *p* = .075, *η²* = .11). Men with ASPD had more cannabis positive tests than women with ASPD and male and female HCs (all *p*’s < .05).

**Tables**

**Table 1**

| **Variable^a^** | **ASPD m** | **(n = 20)** | **ASPD f** | **(n = 18)** | **m** | **HC (n = 20)** | **f** | **HC (n = 20)** | **Effects** |  | ***p* Value** |
| --- | --- | --- | --- | --- | --- | --- | --- | --- | --- | --- | --- |
|  |  |  |  |  |  |  |  |  |  |  |  |
| **Age** | 22.35 | 3.51 | 24.28 | 2.82 | 23.45 | 3.71 | 22.30 | 4.31 | *F*(3,74) = 1.30^b^ | | .286 |
| **IQ (HAWIE-R)** | 103.40 | 13.36 | 103.06 | 11.96 | 105.65 | 15.76 | 103.75 | 15.87 | *F*(3,74) = .13^b^ | | .944 |
|  |  |  |  |  |  |  |  |  |  |  |  |
| **Comorbidities** |  |  |  |  |  |  |  |  |  |  |  |
| **Psychopathy (SRP score > 201)** | 9 | 45.0 | 7 | 38.9 | 0 | 0.0 | 0 | 0.0 | *H*(3) = 21.13^c^ | | <.001 |
| **Borderline Personality Disorder** | 2 | 10.0 | 8 | 44.4 | 0 | 0.0 | 0 | 0.0 | *H*(3) = 21.85^c^ | | <.001 |
| **Past Major Depressive Episode** | 3 | 15.0 | 11 | 61.1 | 0 | 0.0 | 0 | 0.0 | *H*(3) = 30.92^c^ | | <.001 |
| **Alcohol abuse** | 9 | 45.0 | 11 | 61.1 | 0 | 0.0 | 0 | 0.0 | *H*(3) = 29.22^c^ | | <.001 |
| **Cannabis abuse** | 7 | 35.0 | 3 | 16.7 | 0 | 0.0 | 0 | 0.0 | *H*(3) = 14.73^c^ | | .002 |
| **Multiple substance abuse** | 14 | 70.0 | 8 | 44.4 | 0 | 0.0 | 0 | 0.0 | *H*(3) = 34.86^c^ | | <.001 |
| **Posttraumatic Stress Disorder** | 1 | 5.0 | 4 | 22.2 | 0 | 0.0 | 0 | 0.0 | *H*(3) = 10.18^c^ | | .017 |
|  |  |  |  |  |  |  |  |  |  |  |  |
| **Psychometric tests** |  |  |  |  |  |  |  |  |  |  |  |
| **SRP** |  |  |  |  |  |  |  |  |  |  |  |
| **Interpersonal manipulation** | 52.70 | 9.40 | 56.17 | 12.42 | 36.65 | 7.49 | 32.45 | 7.23 | *F*(3,74) = 30.73^b^ | | <.001 |
| **Callous affect** | 47.80 | 6.85 | 44.44 | 12.10 | 38.30 | 4.89 | 29.45 | 6.35 | *F*(3,39.87) = 26.42^b^ | | <.001 |
| **Erratic life style** | 56.00 | 5.89 | 55.28 | 7.22 | 41.80 | 7.55 | 35.60 | 8.99 | *F*(3,74) = 35.51^b^ | | <.001 |
| **Antisocial behavior** | 46.05 | 7.25 | 38.00 | 9.66 | 24.20 | 7.01 | 21.85 | 6.89 | *F*(3,74) = 43.89^b^ | | <.001 |
| **Total** | 202.55 | 21.20 | 193.89 | 30.79 | 140.95 | 19.54 | 119.35 | 19.26 | *F*(3,74) = 13.77^b^ | | <.001 |
| **STAXI^d^** |  |  |  |  |  |  |  |  |  |  |  |
| **State anger** | 18.89 | 6.98 | 20.61 | 8.20 | 12.05 | 2.80 | 11.06 | 1.47 | *F*(3,34.63) = 14.51^b^ | | <.001 |
| **Trait anger** | 21.42 | 5.09 | 28.00 | 5.15 | 15.70 | 3.95 | 14.67 | 3.76 | *F*(3,71) = 33.73^b^ | | <.001 |
| **Anger reaction** | 10.84 | 2.97 | 14.00 | 2.35 | 8.05 | 2.14 | 7.67 | 2.25 | *F*(3,71) = 26.41^b^ | | <.001 |
| **Anger temper** | 10.58 | 2.57 | 14.00 | 3.36 | 7.65 | 2.13 | 7.00 | 1.94 | *F*(3,71) = 28.87^b^ | | <.001 |
| **Anger in** | 19.53 | 4.39 | 19.17 | 4.68 | 14.60 | 3.84 | 14.33 | 3.60 | *F*(3,71) = 8.69^b^ | | <.001 |
| **Anger out** | 18.64 | 4.86 | 22.33 | 5.38 | 12.60 | 4.01 | 11.89 | 2.76 | *F*(3,71) = 24.22^b^ | | <.001 |
| **Anger control** | 19.63 | 4.31 | 15.50 | 5.18 | 22.95 | 4.48 | 23.89 | 3.97 | *F*(3,71) = 13.00^b^ | | <.001 |
|  |  |  |  |  |  |  |  |  |  |  |  |
|  |  |  |  |  |  |  |  |  |  |  |  |

ASPD = Antisocial personality disorder; f = female; HAWIE-R = Wechsler Adult Intelligence Scale – Revised, German version (used for estimation of IQ); HC = healthy control; m = male; STAXI =State-Trait Anger Expression Inventory; SRP =Self-Report Psychopathy Scale.

^a^The numbers are expressed in means and standard deviations, except for comorbidities (numbers and percentage)

^b^One-way ANOVA

^c^Kruskal-Willis H test

^d^Data missing from one man with ASPD and two HC women.

**Table 2**

| **Variable^a^** | **ASPD m (n = 16)** | | | | |  | | **ASPD f (n = 11)** | | | | | |  | | **HC m (n = 14)** | | | | | |  | | **HC f (n = 18)** | | | |  | |  | |  |
| --- | --- | --- | --- | --- | --- | --- | --- | --- | --- | --- | --- | --- | --- | --- | --- | --- | --- | --- | --- | --- | --- | --- | --- | --- | --- | --- | --- | --- | --- | --- | --- | --- |
|  | **Oxytocin** | | | **Placebo** | | | | **Oxytocin** | | | | **Placebo** | | | | **Oxytocin** | | | | **Placebo** | | | | **Oxytocin** | | | | **Placebo** | | | |  |
| **Salivary samples** | |  | |  | |  | |  | |  | |  | |  | |  | |  | |  | |  | |  | |  | |  | |  | |  |
| **Oxytocin^b^** | 3.92 | | 2.17 | | 3.31 | | 1.52 | | 2.23 | | 0.97 | | 2.22 | | 1.15 | | 2.85 | | 2.04 | | 3.65 | | 2.45 | | 2.48 | | 1.47 | | 2.48 | | 1.77 | |
|  |  | |  | |  | |  | |  | |  | |  | |  | |  | |  | |  | |  | |  | |  | |  | |  | |
| **Plasma samples** | |  | |  | |  | |  | |  | |  | |  | |  | |  | |  | |  | |  | |  | |  | |  | |  |
| **Progesterone^c^** |  | |  | |  | |  | | 2.15 | | 3.54 | | 2.43 | | 3.40 | |  | |  | |  | |  | | 0.90 | | 1.48 | | 0.91 | | 1.64 | |
|  |  | |  | |  | |  | |  | |  | |  | |  | |  | |  | |  | |  | |  | |  | |  | |  | |
| **Urinary samples** |  | |  | |  | |  | |  | |  | |  | |  | |  | |  | |  | |  | |  | |  | |  | |  | |
| **Cannabis positive** | 3 | | 15.0 | | 5 | | 25.0 | | 1 | | 5.6 | | 0 | | 0.0 | | 0 | | 0.0 | | 0 | | 0.0 | | 0 | | 0.0 | | 0 | | 0.0 | |

ASPD = Antisocial personality disorder; f = female; HC = healthy control; m = male

^a^The numbers are expressed in means and standard deviations, except for cannabis positive urinary samples (numbers and percentage)

^b^Levels in pg/mL

^c^Levels in ng/mL

**Study design**

The participants were instructed to sit upright with their head tilted backwards before they exhaled and then inhaled deeply while self-administrating 6 puffs of a nasal spray of either OT (24 IU) or PLC, alternating between nostrils (with 45 seconds between puffs). After administration, they were asked to lie down for 10 min.

**fMRI data acquisition and analysis**

Thirty-five 2 mm transverse slices (no gap) were acquired in each volume covering the temporal lobe and occipital and orbitofrontal cortex to achieve high anatomical resolution of the amygdala (TR/TE = 2,260/30 ms, flip angle = 80°, FOV = 208x208 mm, in-plane resolution 2x2 mm). Additionally, a high-resolution T1-weighted coronal-oriented MPRAGE image (1x1x1 mm^3^) was collected for each participant.

Volumes were manually re-oriented to the anterior-posterior commissure line and corrected for slice timing. Images were then realigned to correct for motion, segmented, and co-registered to the mean functional image. Realigned images were spatially normalized to standard MNI template and smoothed with a 4 mm full-width at half-maximum Gaussian filter. Volumes with motion greater than 5 mm/5° and global intensities more than 3 standard deviations from the mean were detected using SPM ART toolbox. Data were excluded from analyses if >25% of volumes per session were detected as outliers. Despiking was completed with interpolation using the ArtRepair toolbox in SPM. Motion parameters were included as regressors in the general linear model design in first level analyses to correct for slow-drift motion.

First-level analysis included repaired pre-processed volumes, six motion parameters, and all six combinations of emotion (fearful, angry, happy) and initial fixation (eyes, mouth) in each session.

**Results**

**Correlations Between Amygdala Activation and Hormonal Parameters Prior to Substance Administration**

There were no correlations between amygdala activation and neither salivary OT nor plasma progesterone levels prior to PLC and OT administration (all correlations |*r*| ≤ .400, *p* > .050).

**Discussion**

The association between response latencies and amygdala activity in ASPD women was specific to angry faces, which are typically considered threat or provocation cues consistent with ASPD women reporting more trait anger, more anger reaction and less anger control in the STAXI.

Individuals with antisocial behaviors have been repeatedly reported to display amygdala hypoactivity especially to fearful faces^5^ which we did not find. However, previous studies used different samples, i.e., adolescents with disruptive behavior disorders and psychopathic traits^6^, adolescents with conduct problems and callous-unemotional traits^7^, adults with the psychopathic subtype of ASPD^5,8,9^ and male participants from a community sample^10^. Notably, the diagnostic category of ASPD is based on behaviors rather than characterological specifiers which interindividually differ between hyperemotional vs. callous-unemotional traits also indicated by the comorbid disorders found in our sample^11,12^.

Previously, it has been reported that OT consistently reduced amygdala activity in response to negative emotional faces, particularly fear, compared with PLC in healthy men^13,14^. In contrast, OT increased amygdala activity in healthy women^15–17^. In a similar way, OT increased amygdala reactivity to threatening scenes in women but not men^18^. Hence, we could replicate the OT effect on amygdala activity in healthy women. The sex differential effects of OT might be explained by OT’s interaction with sex steroid hormones such as progesterone^19^ or baseline sex differences in the neural OT system^20,21^. Our sample showed differences in salivary OT levels with higher levels in women than in men across groups. However, neither peripheral OT levels nor progesterone levels prior to the experiments correlated with amygdala activation following PLC and OT administration. This result could be explained by the finding that a single measurement of peripheral OT is not a reliable trait marker of the physiology of the OT system in humans^22^.

Furthermore, we found that the OT effect on right amygdala activation tended to be generally stronger in women than in men in the total sample, as did the amygdala activation in response to angry faces. The latter trend was also found in the healthy sample. It is of note that the female groups did not differ in progesterone levels which has been shown to modulate amygdala function in women^19^. Sex differences in right amygdala activity during face processing have been reported in a large adolescent sample^23^, namely when angry faces were presented. However, meta-analyses in adults have not confirmed a consistent pattern of sex differences in amygdala activation during emotional faces processing^24^.

We specifically chose the emotion classification task with particularly short presentation times to investigate reflexive processing of facial emotion expressions controlling for the initial fixation on eyes or mouth. Under PLC, we found a stronger anger-related amygdala activation in the ASPD group than HC group when the mouth was initially fixated. This finding is matching previous reports that deficits in facial emotion recognition in children with psychopathic traits can be improved by directing the subject’s attention towards the eyes^25^ leading to the assumption that OT might improve facial emotion recognition by modulating the coupling between amygdala and regions of eye movements targeting attention towards the eye region^26^. Our OT effect on the amygdala was, however, independent of the facial region initially fixated in angry faces. This is consistent with prior findings in healthy individuals^18^. Thus, the emotional processing seemed to be more important for the OT effect than the attentional shift.

**References**

1. Erdfelder, E. & Buchner, A. GPOWER: A general power analysis program. *Behav. Res. Methods, Instruments, Comput.* **28**, 1–11 (1996).

2. Cohen, J. The statistical power of abnormal-social psychological research: A review. *J. Abnorm. Soc. Psychol.* **65**, 145–153 (1962).

3. Bertsch, K. *et al.* Oxytocin and reduction of social threat hypersensitivity in women with borderline personality disorder. *Am J Psychiatry* **170**, 1169–1177 (2013).

4. Timmermann, M. *et al.* Oxytocin improves facial emotion recognition in young adults with antisocial personality disorder. *Psychoneuroendocrinology* **85**, (2017).

5. Blair, R. J. R. The neurobiology of psychopathic traits in youths. *Nature Reviews Neuroscience* vol. 14 786–799 (2013).

6. Marsh, A. A. & Blair, R. J. R. Deficits in facial affect recognition among antisocial populations: A meta-analysis. *Neuroscience and Biobehavioral Reviews* vol. 32 454–465 (2008).

7. Jones, A. P., Laurens, K. R., Herba, C. M., Barker, G. J. & Viding, E. Amygdala hypoactivity to fearful faces in boys with conduct problems and callous-unemotional traits. *Am. J. Psychiatry* **166**, 95–102 (2009).

8. Herpertz, S. C., Bertsch, K. & Jeung, H. Neurobiology of Criterion A: self and interpersonal personality functioning. *Curr. Opin. Psychol.* **21**, (2018).

9. Decety, J., Skelly, L., Yoder, K. J. & Kiehl, K. A. Neural processing of dynamic emotional facial expressions in psychopaths. *Soc. Neurosci.* **9**, 36–49 (2014).

10. Seara-Cardoso, A., Sebastian, C. L., Viding, E. & Roiser, J. P. Affective resonance in response to others’ emotional faces varies with affective ratings and psychopathic traits in amygdala and anterior insula. *Soc. Neurosci.* **11**, 140–152 (2016).

11. Saß, H., Herpertz, S. & Houben, I. Personality disorders: conceptual issues and responsibility. *Jpn. J. Psychiatry Neurol.* **48 Suppl**, 5–17 (1994).

12. Herpertz, S. C. Emotional processing in personality disorder. *Curr. Psychiatry Rep.* **5**, 23–27 (2003).

13. Kirsch, P. *et al.* Oxytocin modulates neural circuitry for social cognition and fear in humans. *J. Neurosci.* **25**, 11489–11493 (2005).

14. Domes, G. *et al.* Oxytocin Attenuates Amygdala Responses to Emotional Faces Regardless of Valence. *Biol. Psychiatry* **62**, 1187–1190 (2007).

15. Tully, J., Gabay, A. S., Brown, D., Murphy, D. G. M. & Blackwood, N. The effect of intranasal oxytocin on neural response to facial emotions in healthy adults as measured by functional MRI: A systematic review. *Psychiatry Res. Neuroimaging* **272**, 17–29 (2018).

16. Rilling, J. K. *et al.* Sex differences in the neural and behavioral response to intranasal oxytocin and vasopressin during human social interaction. *Psychoneuroendocrinology* **39**, 237–248 (2014).

17. Lieberz, J. *et al.* Kinetics of oxytocin effects on amygdala and striatal reactivity vary between women and men. *Neuropsychopharmacology* **45**, 1134–1140 (2020).

18. Lischke, A. *et al.* Oxytocin increases amygdala reactivity to threatening scenes in females. *Psychoneuroendocrinology* **37**, 1431–1438 (2012).

19. Van Wingen, G. A. *et al.* Progesterone selectively increases amygdala reactivity in women. *Mol. Psychiatry* **13**, 325–333 (2008).

20. Borland, J. M., Rilling, J. K., Frantz, K. J. & Albers, H. E. Sex-dependent regulation of social reward by oxytocin: an inverted U hypothesis. *Neuropsychopharmacology* **44**, 97–110 (2019).

21. Winterton, A., Westlye, L. T., Steen, N. E., Andreassen, O. A. & Quintana, D. S. Improving the precision of intranasal oxytocin research. *Nat. Hum. Behav.* **5**, 9–18 (2021).

22. Martins, D., Gabay, A. S., Mehta, M. & Paloyelis, Y. Salivary and plasmatic oxytocin are not reliable trait markers of the physiology of the oxytocin system in humans. *Elife* **9**, 1–19 (2020).

23. Schneider, S. *et al.* Boys do it the right way: sex-dependent amygdala lateralization during face processing in adolescents. *Neuroimage* **56**, 1847–1853 (2011).

24. Fusar-Poli, P. *et al.* Functional atlas of emotional faces processing: A voxel-based meta-analysis of 105 functional magnetic resonance imaging studies. *Journal of Psychiatry and Neuroscience* vol. 34 418–432 (2009).

25. Dadds, M. R. *et al.* Attention to the eyes and fear-recognition deficits in child psychopathy. *Br. J. Psychiatry* **189**, 280–281 (2006).

26. Gamer, M., Zurowski, B. & Büchel, C. Different amygdala subregions mediate valence-related and attentional effects of oxytocin in humans. *Proc. Natl. Acad. Sci. U. S. A.* **107**, 9400–9405 (2010).
