# Supplementary material for: Oxytocin effects on amygdala reactivity to angry faces in males and females with antisocial personality disorder
Source: Neuropsychopharmacology. 2023 Mar 20;48(6):946–53. doi: 10.1038/s41386-023-01549-9 (PMC10156793; doi:10.1038/s41386-023-01549-9)
Supplement: Supplementary file 2 — Supplemental Material 2 [file 41386_2023_1549_MOESM2_ESM.doc]

**
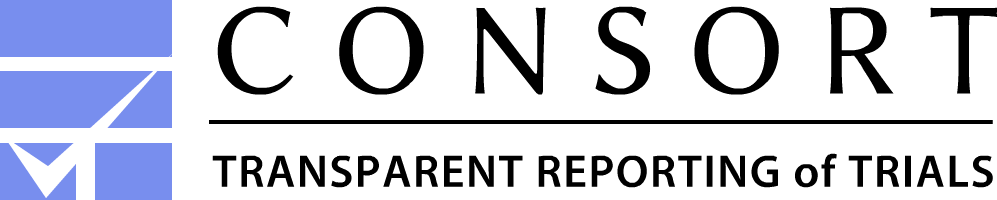
**

**CONSORT 2010 Flow Diagram**

**1st Measurement**

**Analysis**

**2nd Measurement**

**Enrollment**

Assessed for eligibility (n=166)

Excluded (n=64)

♦  Not meeting inclusion criteria (n=24)

♦  Declined to participate (n= 15)

♦  Other reasons (n=25)

Analysed (n=38)
♦ Excluded from analysis (technical artifacts, acute neurological finding) (n=10)

Lost to 2^nd^ measurement (did not show up) (n=3)

ASPD patients (n=51)

♦ Received 1^st^ fMRI measurement (n=51)

Lost to 2^nd^ measurement (did not show up) (n=3)

Healthy controls (n=51)

♦ Received 1^st^ fMRI measurement (n=51)

Analysed (n=40)
♦ Excluded from analysis (technical artifacts) (n=8)

Participants (n=102)
